# SiLRL1, a bHLH transcription factor from foxtail millet, promotes carotenoid accumulation and improves drought tolerance

**DOI:** 10.1186/s12870-025-07825-8

**Published:** 2025-12-06

**Authors:** Yiqiong Huo, Mengdi Wang, Xin Wan, Yiru Zhao, Huarui Han, Bin Zhang, Siyu Hou, Xuemei Ren, Jiayu Song, Yuanhuai Han

**Affiliations:** 1 Shanxi Hou Ji Laboratory, College of Agriculture, Shanxi Agricultural University, Jinzhong, Shanxi 030801 China; 2 Shanxi Hou Ji Laboratory, College of Life Sciences, Shanxi Agricultural University, Jinzhong, Shanxi 030801 China; 3 Institute of Crop and Nuclear Technology Utilization, Zhejiang Academy of Agricultural Sciences, Hangzhou, 310021 China

**Keywords:** Foxtail millet, SiLRL1, Carotenoid, ABA, Drought tolerance, Transcriptome

## Abstract

Foxtail millet, an ancient cereal crop domesticated in China, is valued for its nutritional richness, particularly its carotenoid content. Carotenoids play critical roles in plant development and stress responses, and their metabolic pathways are regulated by various transcription factors (TFs). However, the transcriptional regulatory mechanisms controlling carotenoid accumulation in cereal crops remain poorly understood. In this study, we characterized SiLRL1, a basic helix-loop-helix (bHLH) TF in foxtail millet, and investigated its role in carotenoid metabolism and abiotic stress tolerance. Phylogenetic analysis classified SiLRL1 into subfamily XI of the bHLH family, which lacks a DNA-binding basic region but retains the HLH dimerization domain. Heterologous expression of *SiLRL1* in *Arabidopsis thaliana* significantly enhanced carotenoid accumulation, particularly violaxanthin, and increased abscisic acid (ABA) levels. RNA-seq analysis revealed that *SiLRL1* overexpression downregulated key carotenoid degradation genes (e.g., *AtNCED4*/*CCD4*) and upregulated genes involved in the mevalonate (MVA) and methylerythritol phosphate (MEP) pathways, as well as carotenogenic genes (e.g., *AtPSY*, *AtLCYB*, *AtLCYE*). Additionally, *SiLRL1* overexpression improved drought tolerance and ABA sensitivity in transgenic Arabidopsis, accompanied by altered expression of ABA-responsive and drought-responsive genes. These findings establish SiLRL1 as a positive regulator of carotenoid biosynthesis and drought tolerance, providing insights into the genetic control of carotenoid metabolism and stress adaptation in foxtail millet.

**Supplementary Information:**

The online version contains supplementary material available at 10.1186/s12870-025-07825-8.

## Introduction

 Foxtail millet (*Setaria italica* (L.) Beauv.), one of the world’s most ancient cultivated cereals, was domesticated from green foxtail (*Setaria viridis*) approximately 11,000 years ago [1]. It was first domesticated in China around 8,700 years ago and is now widely cultivated in countries such as India, Nigeria, and the United States, where it is well-adapted to arid and semi-arid environments [2]. After dehulling, the kernal is valued for its high nutritional content, particularly its carotenoids, which represent an important class of nutrients [3, 4].

Carotenoids play essential roles in plant growth and development by acting as light-harvesting pigments and structural components of photosystems [5]. Carotenoid metabolism is a complex process, and structural genes involved in its pathway have been identified in multiple plant species, including *ZmPSY3* in maize [6], *GmCCD4* in soybean [7], *SlCCD1A* in tomato [8], and *DcLCYB2* in carrot [9]. Lycopene serves as a key branching point in the pathway and is cyclized by LCYB (lycopene β-cyclase) and LCYE (lycopene ε-cyclase) to form α-carotene or β-carotene, which are further modified into lutein through hydroxylation or epoxidation [10]. Key enzymes such as PSY (phytoene synthase), CRTISO (carotenoid isomerase), LCYE and LCYB catalyze critical steps and represent major regulatory nodes in carotenoid metabolism [10].

Abscisic acid (ABA), a 15-carbon sesquiterpenoid phytohormone, is derived from the cleavage of β-carotene and plays a central role in enhancing plant tolerance to abiotic stress [11, 12],. ABA biosynthesis begins with the conversion of zeaxanthin to violaxanthin by zeaxanthin epoxidase (ZEP). Violaxanthin then enters one of two pathways: one mediated by neoxanthin synthase (NSY) and an unknown isomerase that converts it to trans-neoxanthin and then to 9’-cis-neoxanthin; the other directly catalyzed by an unidentified isomerase to form 9’-cis-violaxanthin [13]. Both pathways converge at the step catalyzed by 9’-cis-neoxanthin dioxygenase (NCED), which oxidizes these precursors into xanthoxin—the rate-limiting reaction in ABA biosynthesis [14–17]. Additionally, *ABSCISIC ACID DEFICIENT 2* (*ABA2*), which encodes a short-chain dehydrogenase/reductase, is essential for ABA synthesis [18]. *ABSCISIC ACID INSENSITIVE 5* (*ABI5*), a downstream component of ABA signaling, is positively regulated by ABA2 [19], while *ABI3* and *ABI4* also function as major regulators in the ABA signaling cascade [20, 21].

A number of transcription factors (TFs) are known to regulate structural genes in the carotenoid pathway. For example, in citrus, the MADS-box TFs CsMADS5 and CsMADS6 bind directly to the promoters of *PSY* and *PDS* to activate their expression and may form synergistic complexes to enhance carotenoid accumulation [22, 23]. In chrysanthemum, the GARP TF CmUIF1 interacts with MADS-box proteins CmAP3 and CmPI to form a ternary complex (CmAP3-CmPI-CmUIF1) that regulates *CmCCD4a-2* and modulates carotenoid metabolism [24]. Other regulators include SlBBX20, a B-box zinc finger TF that binds the *PSY1* promoter in tomato [25]; CrMYB68, which suppresses *CrBCH2* and *CrNCED5* to inhibit carotenoid synthesis in citrus peel [26]; and AdMYB7, which activates *AdLCY-b* to promote accumulation in kiwifruit [27]. Similarly, MtWP1 upregulates MtLYCe and MtLYCb to modulate carotenoid content in alfalfa flowers [28]. NAC family TFs such as SlNAC4 in tomato [29], CpNAC1 in papaya [30], and FcrNAC22 in kumquat [31] also promote carotenogenesis. The AP2 TF MdAP2-34 elevates carotenoid levels in apple through activation of *MdPSY2-1* [32], and the ERF TF CsERF061 upregulates multiple carotenogenic genes including *PSY1*, *LCYb1* and *CCD1* in citrus [33]. The bZIP TF HY5 enhances carotenoid synthesis by activating *PSY* [34], and the WRKY TF OfWRKY3 positively regulates *OfCCD4* [35]. Furthermore, the HD-ZIP TF CsHB5 interacts with CsbZIP44 to promote ABA-mediated carotenoid biosynthesis in citrus [36].

The bHLH (basic helix-loop-helix) family represents one of the largest and most important TF groups in plants. A typical bHLH domain consists of approximately 60 amino acids, including an N-terminal basic region that facilitates DNA binding to E-box or G-box motifs, and a C-terminal α-helix-loop-α-helix (HLH) motif that mediates dimerization [37]. These TFs function primarily as dimers and participate in diverse processes such as growth, development, light signaling, and stress responses [38–42]. Several bHLH members are implicated in carotenoid metabolism. For instance, PIFs repress *PSY* expression to limit accumulation [43], while CsTT8 positively regulates both the MEP pathway and carotenoid biosynthesis to promote fruit pigmentation in citrus [44].

Despite these advances, most studies on carotenoid metabolism in cereal crops have focused on manipulating biosynthetic enzymes—such as overexpression of bacterial *CrtB* (encoding phytoene synthase) and *CrtI* (encoding carotene desaturase) in maize [45], co-expression with PSY in wheat and rice [46–48], or silencing of endogenous genes like *BCH* in wheat [49]. Extensive studies have demonstrated a strong correlation between the expression of carotenoid structural genes and carotenoid accumulation in plants. Nevertheless, the transcriptional regulatory mechanisms controlling these genes are still not well characterized, and the regulation of carotenoid metabolism in cereal crops remains largely unexplored. In this study, we characterized *SiLRL1*, a bHLH transcription factor gene in foxtail millet, and demonstrated its role in promoting carotenoid accumulation, enhancing ABA sensitivity, and improving drought tolerance. Our results establish SiLRL1 as a positive regulator of carotenoid biosynthesis and drought resistance in plants.

## Materials and methods

### Plant materials and growth conditions

The foxtail millet (Setaria italica) cultivar Jingu 21 (JG21), characterized by its golden kernel color, rich aroma, and high nutritional value, was used in this study. The seeds were provided by the Shanxi Key Laboratory of Minor Crops Germplasm Innovation and Molecular Breeding at Shanxi Agricultural University. Plants were cultivated in the experimental field of Shanxi Agricultural University, located in Taigu, China (37°25′17″ N, 112°34′31″ E), a region with a semiarid climate and an average annual precipitation of less than 450 mm.

Sampling was initiated 30 days after planting (DAP). At the seedling stage (30 DAP), jointing stage (60 DAP), and heading stage (90 DAP), the following tissues were collected: DL1 (newly emerging leaf), DL2 (expanding leaf), DL3 (fully expanded leaf), Q (leaf sheath), J (stem), ASA (tissue around the shoot apex), and G (root). After heading, grains from the middle section of the ear were harvested at three developmental stages: S1 (initial), S3 (middle), and S5 (late stage) [50]. All samples were immediately frozen in liquid nitrogen and stored at −80 °C for subsequent RNA extraction.

### Stress treatments and physiological indicator measurements in Arabidopsis

Sterilized seeds of wild-type (WT) and transgenic Arabidopsis plants ectopically expressing *SiLRL1* were sown on either 1/2 MS medium (control) or 1/2 MS medium supplemented with 0.25 µM/L ABA, and grown at 22 °C for 13 days. Seedlings cultivated on ABA-free 1/2 MS medium were then transplanted into nutrient soil and grown for an additional two weeks. Subsequently, the leaves were sprayed with ABA solutions at concentrations of 0, 50, 75, and 100 µM/L and continuously treated for 10 days. For drought stress treatment, 4-week-old soil-grown plants of both WT and transgenic Arabidopsis were subjected to water deprivation for one week. Following ABA and drought treatments, plant samples were frozen in liquid nitrogen for subsequent analysis.

Malondialdehyde (MDA) and proline (PRO) contents were measured using the MDA Content Assay Kit (AKFA013C, Boxbio, China) and the PRO Content Assay Kit (AKAM003C, Boxbio, Beijing, China), respectively. All experiments were performed with three biological replicates.

### Identification of bHLH family members in foxtail millet and phylogenetic analysis of SiLRL1

The protein sequences of 158 bHLH family genes from Arabidopsis thaliana were obtained from TAIR (https://www.arabidopsis.org/) and used as queries to align against the foxtail millet proteome using TBtools [51], aiming to identify candidate bHLH proteins. Additionally, the canonical HMM profile (PF00010) for the bHLH family was retrieved from the PFAM database (http://pfam.xfam.org/). Candidate proteins were further screened using HMMER 3.0 with an *E*-value cutoff of < 1e⁻⁵, and the presence of complete conserved domains was verified via the NCBI CDD database (https://www.ncbi.nlm.nih.gov/cdd). This process resulted in the identification of 185 bHLH family members in foxtail millet.

To construct a phylogenetic tree, the SiLRL1 (Si1g20740) protein was aligned with its homologs from the following species: *Oryza sativa* (LOC_Os02g35660.1), *Zea mays* (Zm00001eb244380), *Triticum aestivum* (Traes_7AS_D56F0F9E5), *Sorghum bicolor* (Sobic.004g184200), *Glycine max* (Glyma.17g075200), *Setaria viridis* (Sevir.1g203600.1), *Arabidopsis thaliana* (At5g58010.1), *Solanum lycopersicum* (Solyc12T000420.1), *Brassica oleracea var. Oleracea* (Bol045781), and *Daucus carota* (DCAR_019383). The evolutionary tree was constructed with MEGA 11.0 software using the neighbor-joining method under the Poisson model, with bootstrap testing based on 1000 replicates. Multiple sequence alignment was performed using the online tool ClustalX (https://www.genome.jp/tools-bin/clustalw), and the results were visualized with Online Evolview v3 (https://www.evolgenius.info/evolview-v2/#).

### Structural prediction of XⅠ subfamily proteins and haplotype analysis of SiLRL1 in foxtail millet

The secondary and tertiary structures of the bHLH proteins belonging to subfamily XI in foxtail millet and Arabidopsis thaliana were predicted using the Self-Optimized Prediction Method with Alignment (SOPMA, https://npsa.lyon.inserm.fr/cgi-bin/npsa_automat.pl? page=/NPSA/npsa_server.html) [52] and Swiss-Model Homology Modeling (https://swissmodel.expasy.org/), respectively.

Kernel color parameters of 406 conventional foxtail millet varieties, including a* (red/green value), b* (yellow/blue value), and L* (lightness value), were measured using a non-contact spectrophotometer X-Rite VS540 (X-Rite, Grand Rapids, Michigan, USA) [53]. The b* values were subsequently combined with SNP loci of *SiLRL1* from the same set of varieties for haplotype analysis [54] using CandiHap software [55]. Additionally, the potential functional impact of amino acid substitutions in the SiLRL1 protein was predicted with the PPVED program (http://www.ppved.org.cn/index.html) [56].

### Vector construction and transformation

The coding sequence (CDS) of *SiLRL1* was amplified by PCR using primers SiLRL1c-F1/R1 (Supplemental Table S11) with foxtail millet cDNA as template. The resulting fragment was cloned into the modified pCambia1300 plant expression vector at the *Bam*H I and *Sac* I sites under the control of the ubiquitin promoter. The recombinant plasmid was introduced into Agrobacterium tumefaciens strain GV3101 and subsequently transformed into Arabidopsis (Col-0) via the floral-dip method [57]. Positive transformants were selected on hygromycin (35 µg/mL) plates. Genomic DNA was extracted using the cetyltrimethylammonium bromide (CTAB) method and transgenic plants were confirmed by PCR with primers Hyg-F/R (Supplemental Table S11). Abiotic stress tolerance assays were conducted using homozygous T_3_-generation plants.

### Subcellular localization of the SiLRL1

A fusion expression vector, SiLRL1-GFP, was constructed by inserting the cDNA of the *SiLRL1* gene into the pCambia1300 vector. The recombinant plasmid was introduced into Agrobacterium tumefaciens EHA105 via the heat shock method, and positive clones were selected for subsequent infiltration. Transient expression in tobacco leaves was performed through Agrobacterium-mediated transformation. After 48 h of expression, the subcellular localization of SiLRL1 was observed using a laser confocal microscope (Leica STELLARIS 5, Germany). The nuclear marker H2B-RFP was co-expressed to indicate nuclear compartments. GFP and RFP signals were detected under 488 nm and 552 nm excitation wavelengths, respectively.

### Transcriptome analysis of SiLRL1 transgenic plants

Total RNA was extracted from immature fruit pods of Arabidopsis thaliana (with three biological replicates per line) using the RNApure Plant Kit (DNase I) (CWBIO, Jiangsu, China). cDNA library construction was conducted by Biomarker Biotechnology Company (Beijing, China). Sequencing was carried out on the Illumina NovaSeq 6000 platform. Raw reads were processed to remove adapter sequences, reads containing ambiguous bases (N), and low-quality reads. The quality of the clean data was assessed by calculating Q20, Q30 scores, and GC content. Libraries were pooled according to effective concentration and target data volume for subsequent Illumina sequencing. After quality control, clean reads were aligned to the reference genome (Arabidopsis_thaliana.TAIR10.1.genome.fa) using HISAT2 (v2.2.6) [58]. Transcriptome assembly and reconstruction were performed with StringTie (v1.3.6) [59].

Gene function was annotated against multiple databases, including: NCBI non-redundant protein (Nr) and nucleotide (Nt) sequences, Protein family (Pfam), Clusters of Orthologous Groups of Proteins (KOG/COG), Swiss-Prot, KEGG Ortholog (KO), and Gene Ontology (GO). Gene expression levels were normalized using FPKM (fragments per kilobase of transcript per million mapped reads). Differential expression analysis across the three lines was conducted with the DESeq2 R package (v1.20.0) [60], applying the Benjamini–Hochberg method for false discovery rate (FDR) adjustment. Genes with |log₂FoldChange| >1 and FDR < 0.05 were identified as differentially expressed genes (DEGs). GO enrichment analysis of DEGs was performed using the Cluster Profiler R package with gene length bias correction. Enrichment of KEGG pathways was also analyzed using Cluster Profiler to identify significantly enriched metabolic and signal transduction pathways.

### Gene expression analysis by qRT-PCR

The qRT-PCR primers for genes involved in the carotenoid metabolic pathway were designed using Primer 5.0 (Supplemental Table S11). Si9g11550 (*SiActin*) was used as an internal reference gene. qRT-PCR was performed in triplicate using SYBR Premix Ex Taq II (TaKaRa, RR820A) on a CFX96 Real-Time System (Bio-Rad). Gene expression levels were quantified using the 2^−ΔΔCt^ method.

### Extraction of total carotenoid and UHPLC analysis of carotenoid monomers

Total carotenoid extraction was performed following the methodology outlined by the American Association of Cereal Chemists (AACC) [50, 61]. Analysis of carotenoid monomers was performed on a Thermo DGLC dual ternary ultra-high performance liquid chromatography (UHPLC) system (Vanquish, Thermo, USA) equipped with a DAD detector. Separation was achieved using a YMC Carotenoid S-3 μm column (150 × 4.6 mm) maintained at 40℃. The injection volume was 2 µL, and detection was carried out at a wavelength of 450 nm. The mobile phase consisted of solvent A (methanol) and solvent B (a mixture of methanol, MTBE and water in a ratio of 20:75:5, v/v/v). A constant flow rate of 1.0 ml/min was applied with the following gradient program: 0 min, A:B (100:0, v/v); 15 min, A:B (39:61, v/v); 25 min, A:B (0:100, v/v); 25.1 min, A:B (100: 0, v/v); and 30 min, A:B (100:0, v/v). Throughout the analysis, samples were kept at 4℃ in the autosampler. To minimize the impact of instrumental signal fluctuations, samples were analyzed in randomized order. Quality control (QC) samples were inserted into the sample sequence to monitor system stability and ensure the reliability of the experimental data.

### Hormone quantification methodology

Hormone content analysis was performed using UHPLC system (Vanquish, Thermo, USA) coupled with a high-resolution mass spectrometer (Q Exactive, Thermo, USA).

#### Liquid chromatography parameters

Chromatographic separation was performed on a Waters HSS T3 column (50 × 2.1 mm, 1.8 μm) maintained at 40 °C. The mobile phase comprised solvent A (ultrapure water with 0.1% acetic acid) and solvent B (acetonitrile with 0.1% acetic acid), delivered at a flow rate of 0.3 ml/min. The injection volume was 2 µL. The gradient elution program was set as follows: 0 min, A:B (90:10, v/v), 1 min, A:B (90:10, v/v), 5 min, A:B (10:90, v/v), 7 min, A:B (10:90, v/v), 7.1 min, A:B (90:10, v/v) and 9 min, A:B (90:10, v/v). Throughout the analysis, samples were maintained at 4 °C in the autosampler. To mitigate the impact of instrumental signal fluctuations, samples were analyzed in randomized order. QC samples were inserted into the sequence to monitor system stability and ensure data reliability.

#### Mass spectrometry parameters

Mass spectrometric detection was carried out using a Q Exactive high-resolution mass spectrometer (Thermo, USA) equipped with an electrospray ionization (ESI) source operating in negative ion mode. The ESI parameters were set as follows: sheath gas flow, 40 arb; auxiliary gas flow, 10 arb; ion spray voltage, −2800 V; vaporizer temperature, 350 °C; and ion transfer tube temperature, 320 °C. Data acquisition was performed in selected ion monitoring (SIM) mode with a mass scan range of m/z 100–500.

### Data resource collection

The datasets utilized in this study were obtained from five major sources. Protein sequences of 158 AtbHLHs and 185 SibHLHs (Supplementary Table S2) were acquired from the TAIR database (https://www.arabidopsis.org/) and the MDSi database (http://foxtail-millet.biocloud.net/home), respectively. LRL1 sequences from Arabidopsis thaliana, soybean, rice, maize, and other species (Supplementary Table S5) were retrieved from the NCBI (https://www.ncbi.nlm.nih.gov/) and Phytozome (https://phytozome-next.jgi.doe.gov/). Tissue-specific expression profiles of the 185 *SibHLHs* in 28 different tissues of Yugu1 (Supplementary Table S6) were obtained from the *Setaria* DB (http://111.203.21.71:8000/multi-omics/index.html).

### Statistical analysis

All experiments were performed with three biological replicates, each consisting of three technical replicates. Statistical analysis and data visualization were conducted using GraphPad Prism 7.0. Gene expression heatmaps were generated with the OmicShare toolbox (https://www.omicsmart.com/) [62].

## Results

### Phylogenetic analysis, structural characterization, and haplotype analysis of SiLRL1

**Fig. 1:**
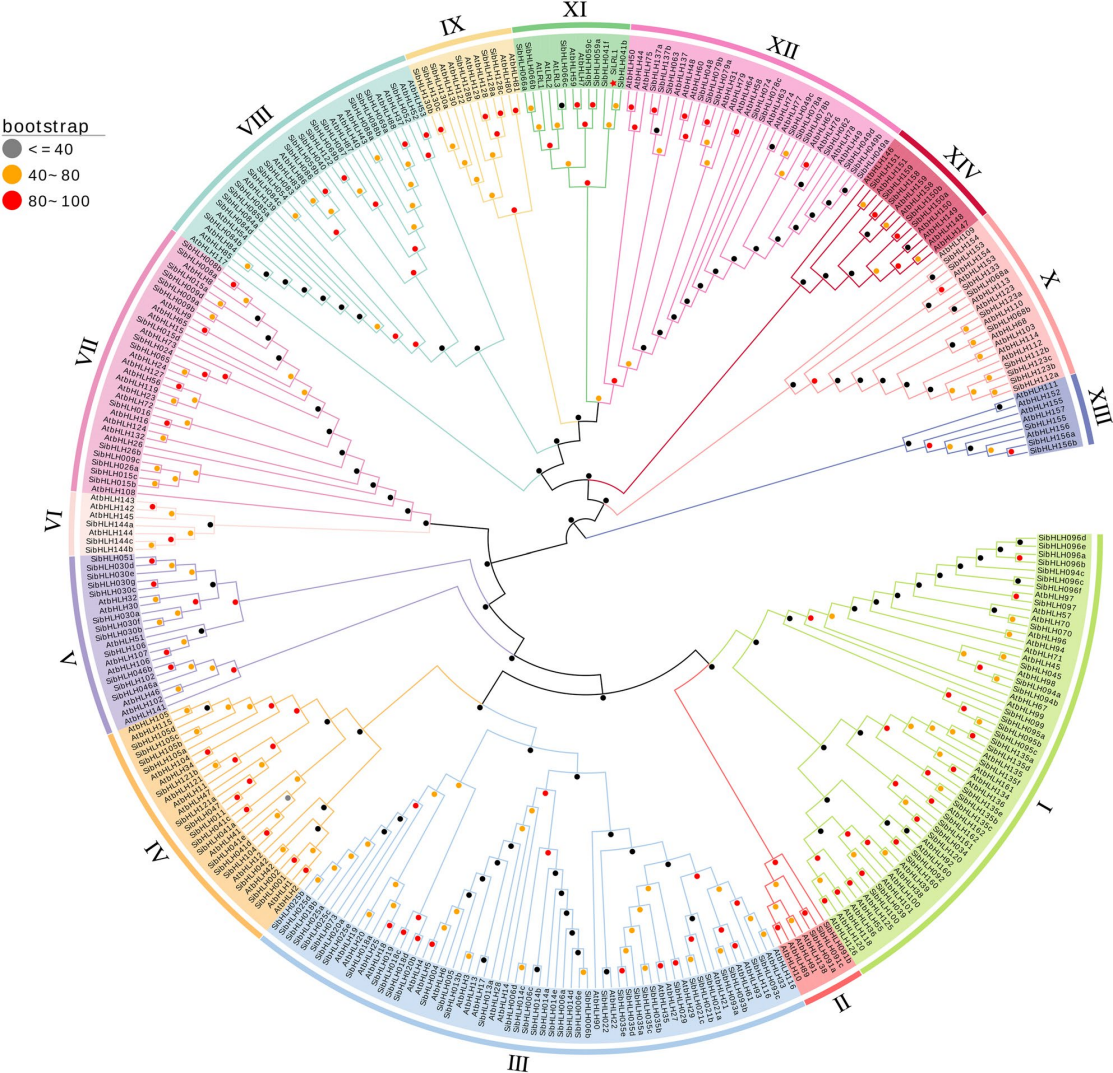
Phylogenetic tree of the bHLH family relationship between *Setaria italica* and *Arabidopsis thaliana*. The tree was derived using the NJ method in MEGA 11.0 and shows 14 phylogenetic subfamilies. The protein sequence of genes used for the phylogenetic tree are shown in Supplemental Table S3

To investigate the evolutionary relationships of bHLH family genes between foxtail millet and Arabidopsis, a phylogenetic tree was constructed using 158 *A. thaliana* and 185 *S. italica* bHLH protein sequences with MEGA 10 software (Supplemental Tables S1 and S2). To ensure analytical reliability, the presence of conserved superfamily domains was verified using the NCBI Conserved Domain Database (CDD). The protein sequences used for phylogenetic reconstruction are provided in Supplemental Table S3. Based on the classification established for Arabidopsis [63], the foxtail millet *bHLH* genes were categorized into 14 groups, designated I to XIV (Fig. 1). Among these, groups Ⅱ, Ⅵ, and ⅩⅢ contained the fewest *SibHLH* members, each with only three genes, followed by group XⅣ with five members. *SiLRL1* belongs to group XⅠ, which comprises eight genes from foxtail millet and five from Arabidopsis (Fig. 1).

**Fig. 2:**
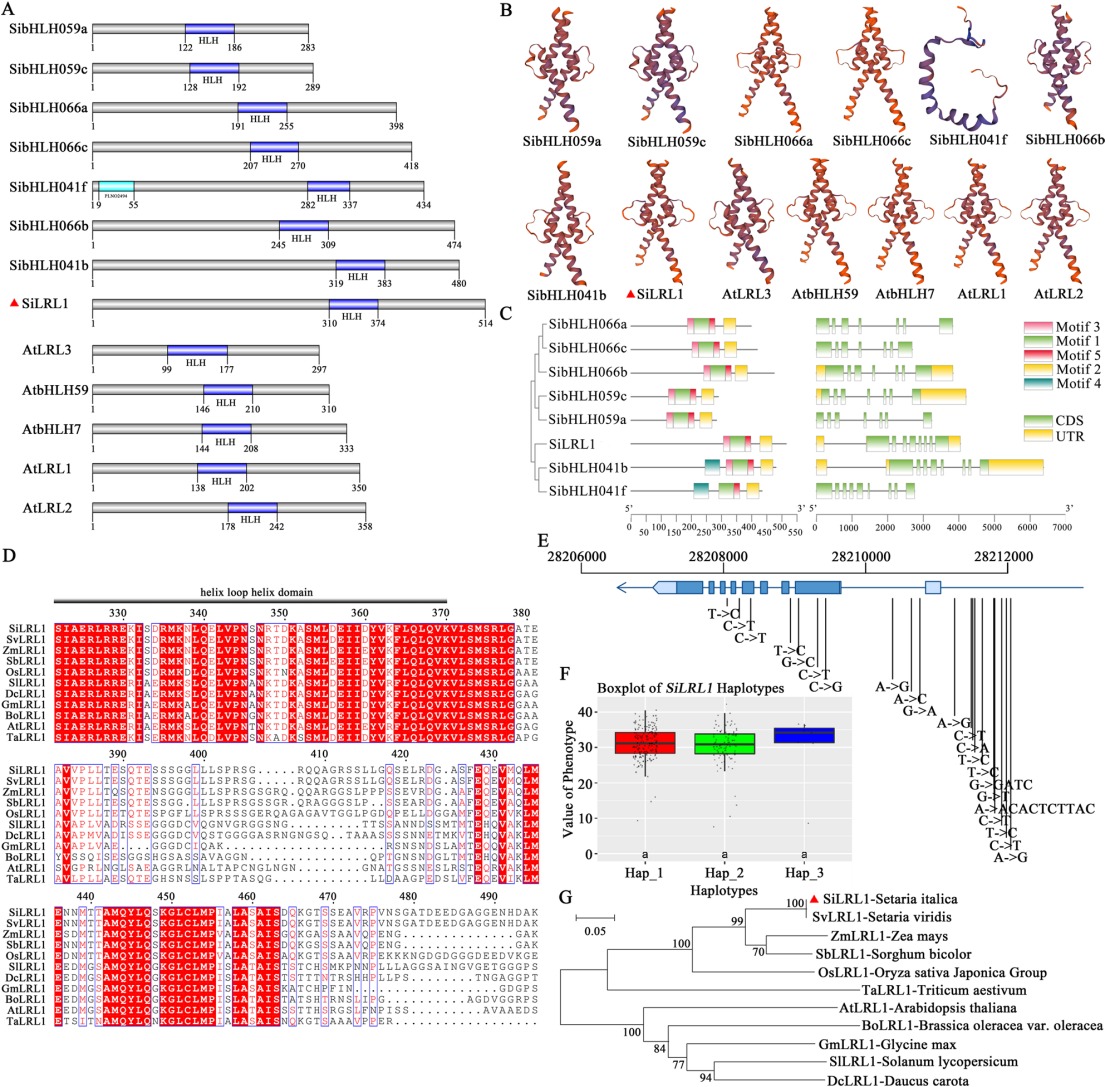
Homology and haplotype analysis of SiLRL1. **A**. XⅠ subfamily domain of the bHLH family in foxtail millet and Arabidopsis. **B**. Tertiary structure prediction model of proteins of XⅠ subfamily in foxtail millet and Arabidopsis. Information of genes and proteins of the bHLH members of XⅠ subfamily is shown in Supplemental Table S4. **C**. Analysis of phylogenetic tree, gene structure, and motif of 8 SibHLHs in the XⅠ subfamily. **D**. Alignment of the putative amino acid segment sequence of SiLRL1 with the amino acid sequences of homologous proteins. bHLH domains were labeled with black lines. **E**. *SiLRL1* haplotype analysis of SNP loci in 406 germplasm resources of foxtail millet. **F**. Boxplot of *SiLRL1* haplotypes. Three main haplotypes of *SiLRL1* were identifed among 406 germplasms. Lowercase letters indicate significant difference (P<0.05, Tukey’s multiple comparisons test). **G**. Phylogenetic tree of *SiLRL1* and homologous genes in some other plant species

All members of the XⅠ subfamily contain a bHLH domain (Fig. 2 A). Among the 13 proteins, 12 exhibit similar tertiary structures, with the exception of SibHLH041f (Fig. 2B). Their secondary structures consist of α-helices, random coils, and extended strands, as detailed in Supplemental Table S4. In SiLRL1, SibHLH066a, SibHLH041f, SibHLH041b, SibHLH066c, and AtLRL1 (AtbHLH66), random coils account for 70% to 80% of the sequences, whereas SibHLH066b, SibHLH059a, SibHLH059c, AtLRL3, and AtbHLH7 contain 30% to 40% α-helices. We further analyzed the eight SibHLH proteins in subfamily XⅠ by examining their phylogeny, gene structure, and motifs. This analysis revealed that motif 1, motif 2, and motif 5 are conserved across all members (Fig. 2 C, Supplemental Figure S2).

To elucidate the evolutionary relationships within the LRL1 gene family, a phylogenetic tree was generated using MEGA 11.0 with protein sequences from 11 species, including foxtail millet, maize, sorghum, rice, and wheat (Supplemental Table S5). The resulting phylogeny indicated that SiLRL1, SvLRL1, ZmLRL1, and SbLRL1 form one clade, while OsLRL1 and TaLRL1 are closely related, and AtLRL1, BoLRL1, GmLRL1, SlLRL1, and DcLRL1 constitute another distinct cluster (Fig. 2G). This grouping suggests that LRL1 from monocotyledonous and dicotyledonous plants diverged significantly, with monocot LRL1s forming one evolutionary group and dicot LRL1s another. Multiple sequence alignment showed that the bHLH domain of SiLRL1 is conserved across all examined species (Fig. 2D).

Haplotype analysis of *SiLRL1* was performed across 406 conventional foxtail millet germplasm accessions to assess potential correlations between genetic variation in *SiLRL1* and kernel color. A total of 22 variant sites were identified within the *SiLRL1* genomic sequence, defining 62 haplotypes (Supplemental Table S7). Among these, four variants were located in exonic regions (Table 1). Only one SNP (4: 28209067, for chromosome 4, position 28,209,067 bp) resulted in an amino acid substitution (N192K); functional impact prediction suggested that this mutation is neutral. Three major haplotypes were identified: Hap_1 (172 varieties), Hap_2 (101 varieties), and Hap_3 (46 varieties) (Table 1, Fig. 2E). However, no significant differences in kernel color were associated with these haplotypes (Fig. 2 F).

**Table 1:**
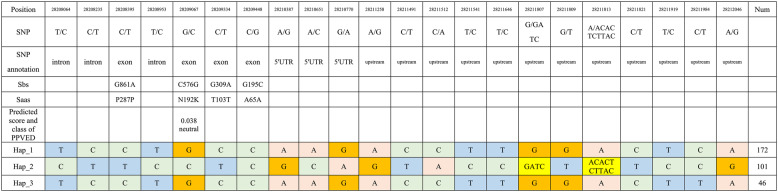
*SiLRL1* haplotype analysis from a core collection of 406 conventional foxtail millet varieties

Three main haplotypes of *SiLRL1* were identifed. Nucleotide variations and number of resources were shown for each haplotype. Sbs, the single base substitution; Saas, the single amino acid substitution. Num, number of varieties. PPVED, plant protein variation effect detector, a tool that predicts the effect of Saas on protein function in plants [56]. Predicted score: the predicted probability score, the value is between 0–1. Predicted class: the predicted binary classification (functional: score >= 0.5; neutral: score <0.5)

### Expression profiling and subcellular localization of SiLRL1 in foxtail millet

**Fig. 3:**
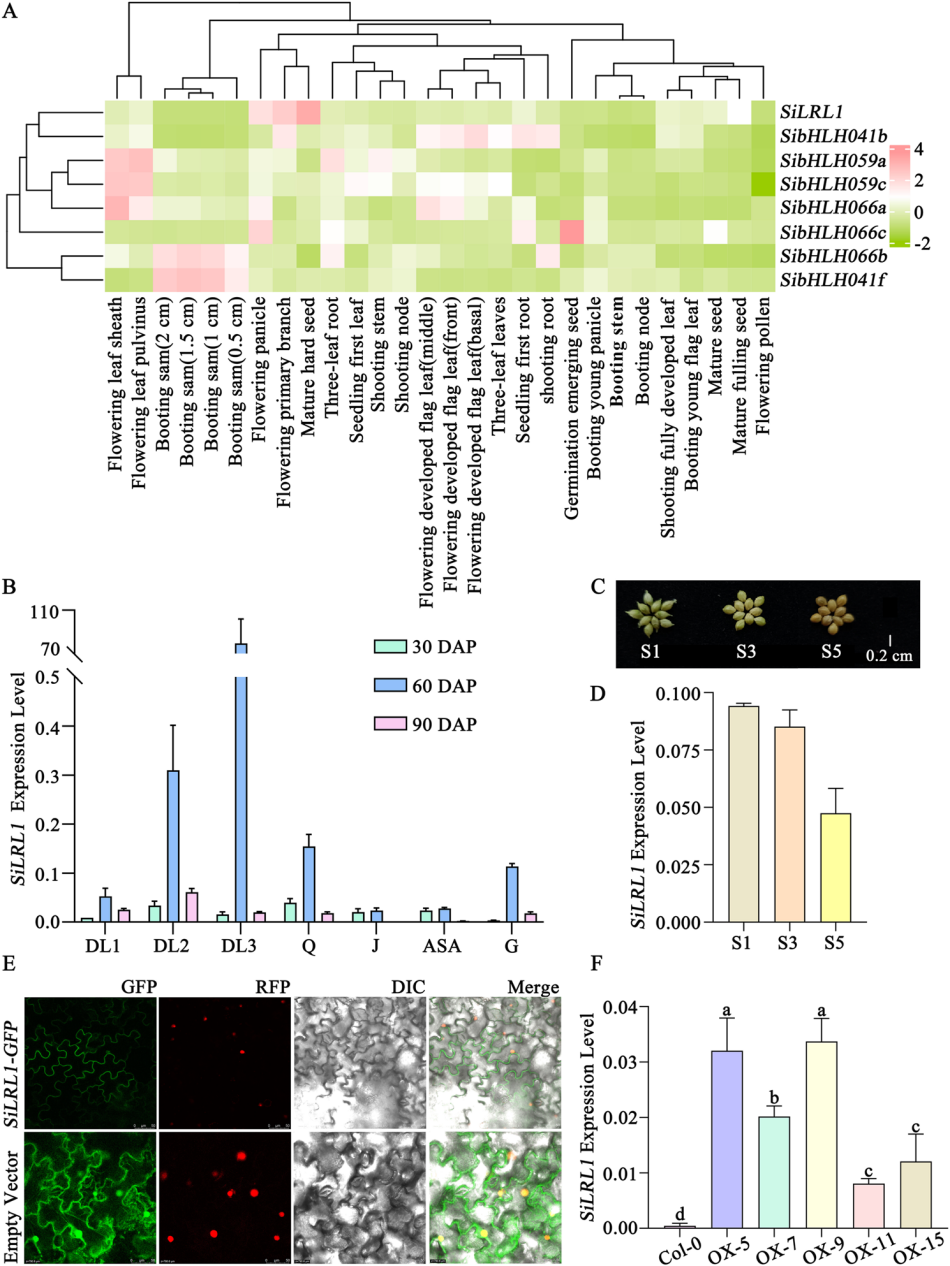
*SiLRL1* expression pattern and protein subcellular localization. **A**. Expression heatmap of the XⅠ subfamily genes of *SibHLHs* in Yugu1. **B** and **D**. Expression pattern of *SiLRL1* in JG21. DL1, newly emerging leaf; DL2, expending leaf; DL3, fully expended leaf; Q, leaf sheath; J, stem; ASA, around the shoot apex; G, root; S1, S3, and S5, the initial stage, middle stage, and last stage of the grain development period. 30 DAP, the seedling stage; 60 DAP, the jointing stage; 90 DAP, the heading stage. **C**. Phenotypic differences in grains of JG21 at S1, S3 and S5. **E**. Subcellular localization of SiLRL1 protein in tobacco epidermal cells. Confocal micrographs show cells expressing either the SiLRL1-GFP fusion protein (upper panels) or free GFP (lower panels). For each construct, the following images are presented: green fluorescence (GFP), red fluorescence from the nuclear marker H2B-RFP (RFP), differential interference contrast (DIC), and a merged image of the GFP, RFP and DIC channels. Scale bars, 50 μm. **F**. *SiLRL1* expression of Col-0 and five *SiLRL1* heterologous expression lines of Arabidopsis (OX-5, OX-7, OX-9, OX-11 and OX-15)

We characterized the expression patterns of 185 *SibHLHs* across 28 different tissues of foxtail millet cultivar Yugu1 (Supplemental Table S6 and Supplemental Figure S1). The results demonstrate that genes from all 14 subgroups display differential expression levels among various tissues. A heatmap focusing on the eight *SibHLH* genes within subfamily XⅠ further revealed divergent expression abundances, suggesting their potential roles in diverse physiological and developmental processes in foxtail millet (Fig. 3 A).

To characterize the expression pattern of *SiLRL1* in the JG21 variety, three growth stages were examined: seedling (30 DAP), jointing (60 DAP), and heading (90 DAP), along with three grain development stages (S1, S3, S5) (Fig. 3B-D). *SiLRL1* expression was significantly higher during the jointing stage, particularly in the leaves (DL3), where it was approximately 5000-fold and 4000-fold greater than that at the seedling and heading stages, respectively (Fig. 3B). Additionally, *SiLRL1* was broadly expressed across multiple tissues at the jointing stage compared to other developmental periods. In developing grains, transcript levels of *SiLRL1* progressively declined during panicle maturation (Fig. 3D).

To determine the subcellular localization of SiLRL1, a SiLRL1-GFP fusion construct and a nuclear marker (H2B-RFP) were co-transfected into 4–5 week-old tobacco epidermal cells via Agrobacterium-mediated transient transformation. While free GFP fluorescence was distributed throughout the cell, the SiLRL1-GFP signal was specifically localized to the cell membrane (Fig. 3E).

### The effect of *SiLRL1* heterologous overexpression on the transcriptomic profiles in *Arabidopsis thaliana*

**Fig. 4:**
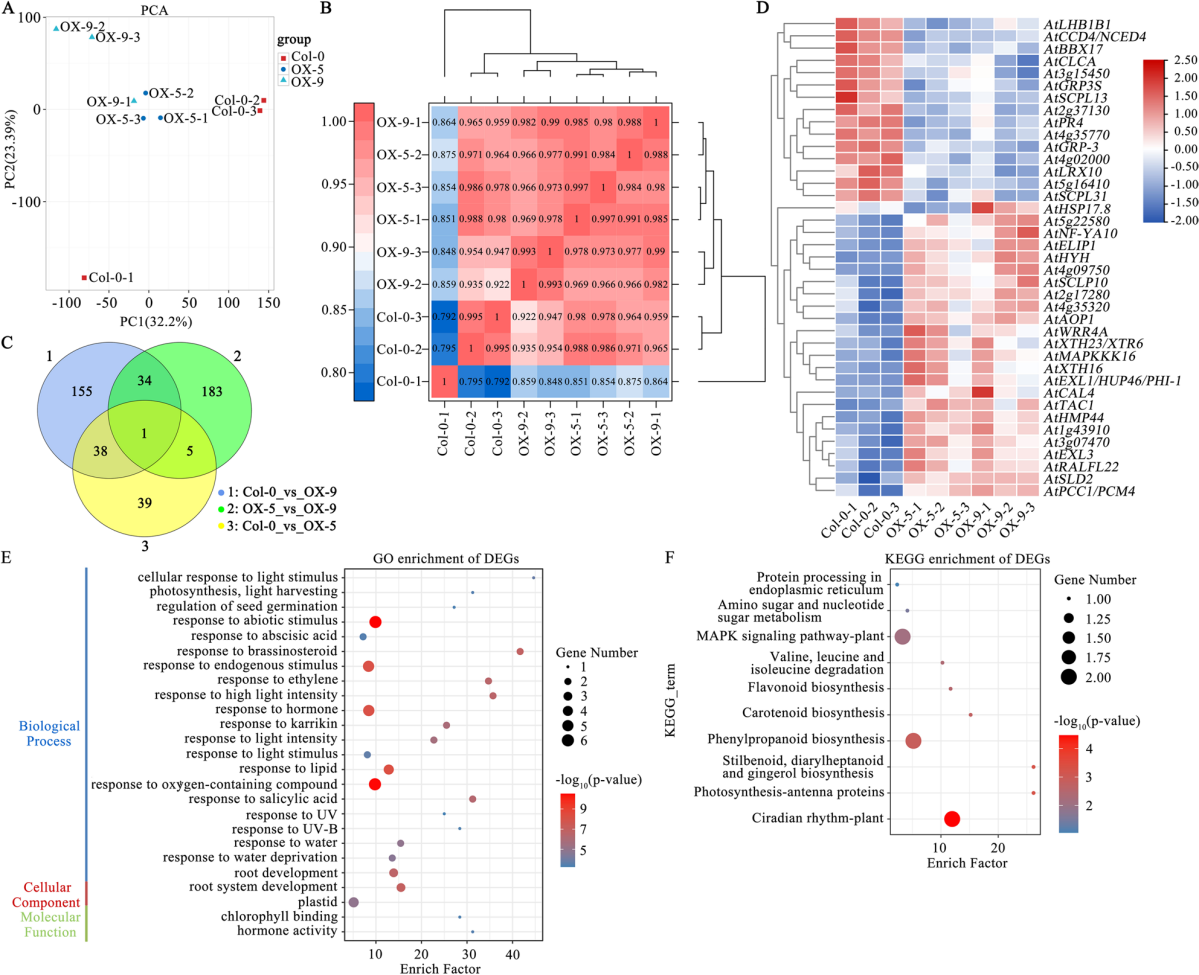
RNA-seq analysis of *SiLRL1* heterologous expression of *Arabidopsis thaliana*. **A**. The principal component analysis (PCA) of the 9 samples. **B**. Correlation analysis of the 9 samples. **C**. Numbers of up- and down-regulated DEGs in different comparison groups. **D**. Heatmap showing the expression patterns of the 39 common DEGs across all samples. The X-axis represents the name of the sample and the clustering results of the sample, whereas the Y-axis represents the clustering results of different genes. Different columns in the figure represent different samples, and different rows represent different genes. The colors show the levels of gene expression in the sample. **E** and **F**. Functional enrichment analysis of the 39 common DEGs. E, Significantly enriched GO terms. F, Significantly enriched KEGG pathways. Each circle represents the GO and KEGG pathway, respectively (names are shown on the left). The size of the circle represents the number of genes and the color gradient represents the extent of enrichment

To investigate the molecular impact of *SiLRL1* overexpression on the transcriptomic profiles in Arabidopsis, we first quantified its transcript levels in multiple transgenic Arabidopsis lines via qRT-PCR. The lines exhibiting the highest expression, OX-5 and OX-9, were selected for subsequent RNA-seq analysis (Fig. 3 F). A total of 57.62 Gb of clean reads were obtained, with Q30 scores exceeding 92.93%, and were aligned to the *Arabidopsis thaliana* reference genome (Supplemental Table S8). The alignment efficiency ranged from 96.55% to 98.19%. Principal component analysis (PCA) and correlation assessment revealed clear transcriptomic divergence between the SiLRL1-overexpressing lines and wild type plants (Fig. 4A-B).

Among the 28,149 genes detected, 456 were differentially expressed (DEGs) in transgenic lines compared to the wild type (Fig. 4 C). Specifically, 83 DEGs were identified in Col-0 vs. OX-5 (44 upregulated, 39 downregulated), and 223 DEGs in Col-0 vs. OX-9 (207 upregulated, 16 downregulated). Further analysis identified 39 common DEGs across comparisons, comprising 24 upregulated and 15 downregulated genes (Fig. 4D, Supplemental Table S9).

GO annotation categorized the DEGs into three functional classes: biological process, cellular component, and molecular function (Fig. 4E, Supplemental Table S9). Within molecular function, 11 genes were significantly enriched (FDR < 0.05), including six involved in enzyme activity regulation: *AtCCD4*/*NCED4* (oxidoreductase); *AtSCPL10*, *AtSCPL13*, *AtSCPL31* (serine-type carboxypeptidases); *AtXTH23*/*XTR6* and *AtXTH16* (hydrolases). *AtRALFL22* was implicated in hormone and receptor activity, while *AtPR4* and *AtLHB1B1* were associated with chitin and chlorophyll binding. In biological processes, DEGs were significantly enriched in 81 GO terms, 34 of which were related to responses to abiotic stimuli such as light, UV, water, low temperature, chemicals, drugs, and alcohol. Cellular component analysis indicated enrichment in the cell wall (e.g., *AtEXL3*, *AtXTH23/XTR6*, *AtXTH16*, *AtEXL1/HUP46/PHI-1*, *AtRALFL22*), plastids (e.g., *AtXTH23/XTR6*, *AtXTH16*, *AtRALFL22*, *AtELIP1*, *AtLHB1B1*), and photosystems (*AtLHB1B1*).

KEGG pathway enrichment analysis revealed that 12 DEGs were assigned to 10 significantly enriched metabolic pathways (FDR < 0.05)(Fig. 4 F, Supplemental Table S9), including carotenoid biosynthesis (*AtNCED4*/*CCD4*), flavonoid biosynthesis (*At5g16410*), circadian rhythm (*AtHYH*, *AtBBX17*, *AtPCC1*/*PCM4*), phenylpropanoid biosynthesis (*At5g16410*, *At2g37130*), and the MAPK signaling pathway (*AtMAPKKK16*, *AtPR4*). Genes involved in circadian rhythm and amino acid degradation were upregulated, whereas *AtCCD4*/*NCED4*—a key gene in carotenoid degradation—was downregulated, suggesting a potential role for *SiLRL1* in inhibiting carotenoid degradation.

### Regulation of carotenoid metabolism pathway genes in Arabidopsis by *SiLRL1*

**Fig. 5:**
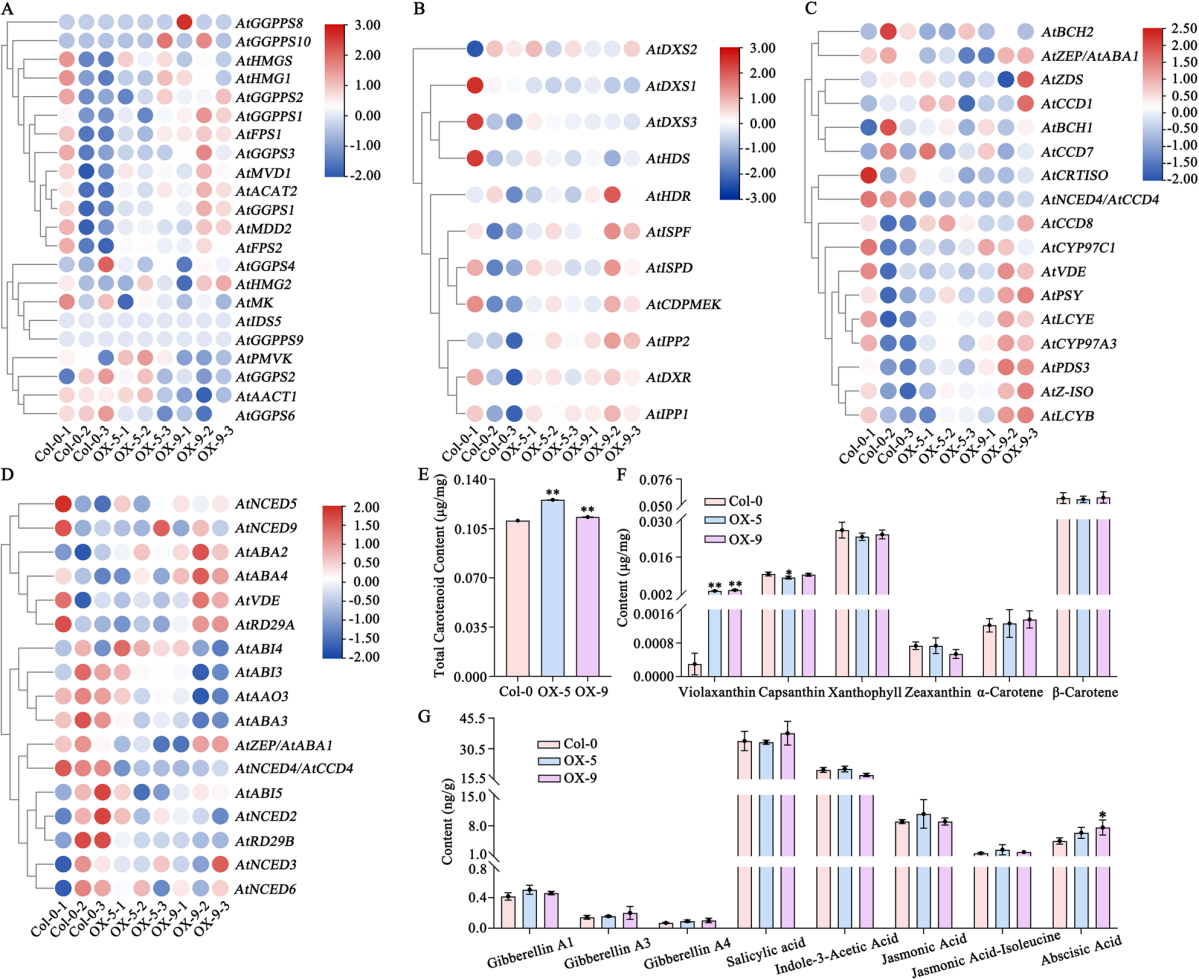
Differences of gene expression of carotenoid pathway, carotenoid content, and hormone content in Col-0, OX-5 and OX-9. **A**-**D**. Expression heatmap of genes of MVA pathway (A), MEP pathway (B), carotenoid synthesis pathway (C) and ABA pathway (D). **E**. Total carotenoid content. **F**. Carotenoid monomer content. **G**. Hormone content

To elucidate the role of *SiLRL1* in carotenoid metabolism, we analyzed the expression profiles of carotenoid pathway-related genes in the *SiLRL1*-overexpressing lines OX-5 and OX-9. In the MVA pathway, 11 genes—*AtHMGS*, *AtHMG1*, *AtGGPPS2*, *AtGGPPS1*, *AtFPS1*, *AtGGPS3*, *AtMVD1*, *AtACAT2*, *AtGGPS1*, *AtMDD2* and *AtFPS2*—exhibited higher expression in transgenic lines compared to Col-0, whereas *AtGGPS6* and *AtGGPS2* were downregulated (Fig. 5A; Supplemental Table S10). No expression was detected for *AtGGPPS8*, *AtGGPPS9*, *AtGGPPS10* or *AtIDS5* in either wild-type or transgenic plants. Similarly, most genes in the MEP pathway—including *AtDXR*, *AtIPP2*, *AtIPP1*, *AtHDR*, *AtISPF*, *AtISPD* and *AtCDPMEK*—were upregulated slightly in the transgenic lines (Fig. 5B; Supplemental Table S10).

Furthermore, the majority of key carotenogenic genes showed varying degrees of upregulation in transgenic plants (Fig. 5C; Supplemental Table S10). Elevated expression of upstream genes in the carotenoid synthesis pathway such as *AtPSY*, *AtPDS3* and *AtZ-ISO* might lead to increased accumulation of phytoene, phytofluene, and ζ-carotene. Upregulation of *AtLCYE* and *AtLCYB* in the α- and β-branch synthesis pathways resulted in modest increases in α-carotene and β-carotene content (Fig. 5C, F). *AtCYP97A3* and *AtCYP97C1* were slightly upregulated, coinciding with minor changes in zeaxanthin and xanthophyll levels (Fig. 5F). In contrast, *AtNCED4*/*AtCCD4* was significantly downregulated (Fig. 5C). These results indicate that *SiLRL1* overexpression enhances the expression of multiple carotenogenic genes, although some genes were suppressed.

**Fig. 6:**
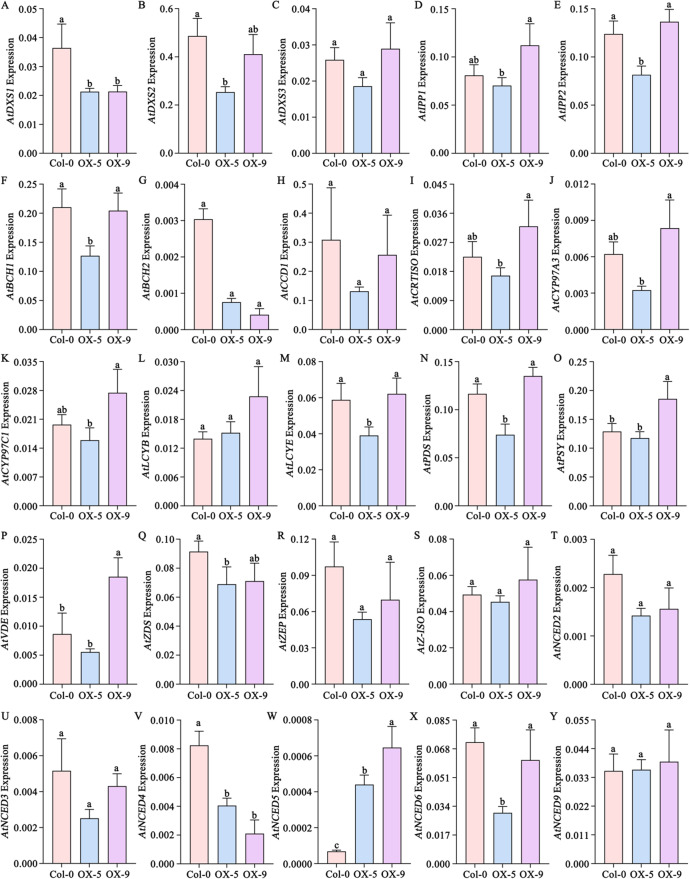
Expression of carotenoid metabolic pathway genes in Col-0, OX-5 and OX-9. Lowercase letters indicate significant difference (P<0.05, Tukey’s multiple comparisons test). A-C. AtDXSs. D-E. AtIPPs. F-G. AtBCHs. H. AtCCD1. I. AtCRTISO. J. AtCYP97A3. K. AtCYP97C1. L. AtLCYB. M. AtLCYE. N. AtPDS. O. AtPSY. P. AtVDE. Q. AtZDS. R. AtZEP. S. AtZ-ISO. T-Y. AtNCEDs

Altered expression was also observed in downstream carotenoid metabolic pathways. The ABA biosynthetic genes *AtABA2* and *AtABA4* were upregulated, whereas *AtAAO3*—which catalyzes the final step of ABA synthesis—was downregulated, along with ABA signaling genes *AtABI3* and *AtABI4* (Fig. 5D; Supplemental Table S10). Additionally, key ABA synthesis enzymes including *AtNCED2*, *AtNCED4* and *AtNCED5* showed reduced expression (Fig. 5D). Despite these transcriptional changes, ABA content was significantly higher in OX-9 than in the wild type (Fig. 5G). To corroborate the transcriptome findings, qRT-PCR was performed on selected carotenoid metabolic genes(Fig. 6). The expression trends were consistent with the RNA-Seq data, confirming the reliability of the transcriptome analysis.

### Heterologous expression of *SiLRL1* promoted carotenoid and ABA accumulation, and enhanced drought tolerance and ABA sensitivity in Arabidopsis

To determine whether SiLRL1 influences carotenoid accumulation, we quantified total carotenoid levels and specific carotenoid monomers in immature siliques of two heterologous expression lines (OX-5, OX-9) and Col-0. Total carotenoid content was significantly higher in both transgenic lines compared to Col-0 (*p* < 0.01; Fig. 5E). Violaxanthin levels were also markedly elevated in OX-5 and OX-9, while capsanthin, xanthophyll, zeaxanthin, α-carotene, and β-carotene showed no significant changes (Fig. 5 F). We next analyzed hormone levels in immature siliques. Gibberellins (GA1, GA3, GA4), salicylic acid (SA), indole-3-acetic acid (IAA), jasmonic acid (JA), jasmonic acid-isoleucine (JA-Ile), and ABA were quantified. Among these, only ABA content was significantly increased in OX-9 compared to Col-0; other hormones exhibited no notable differences (Fig. 5G).

**Fig. 7:**
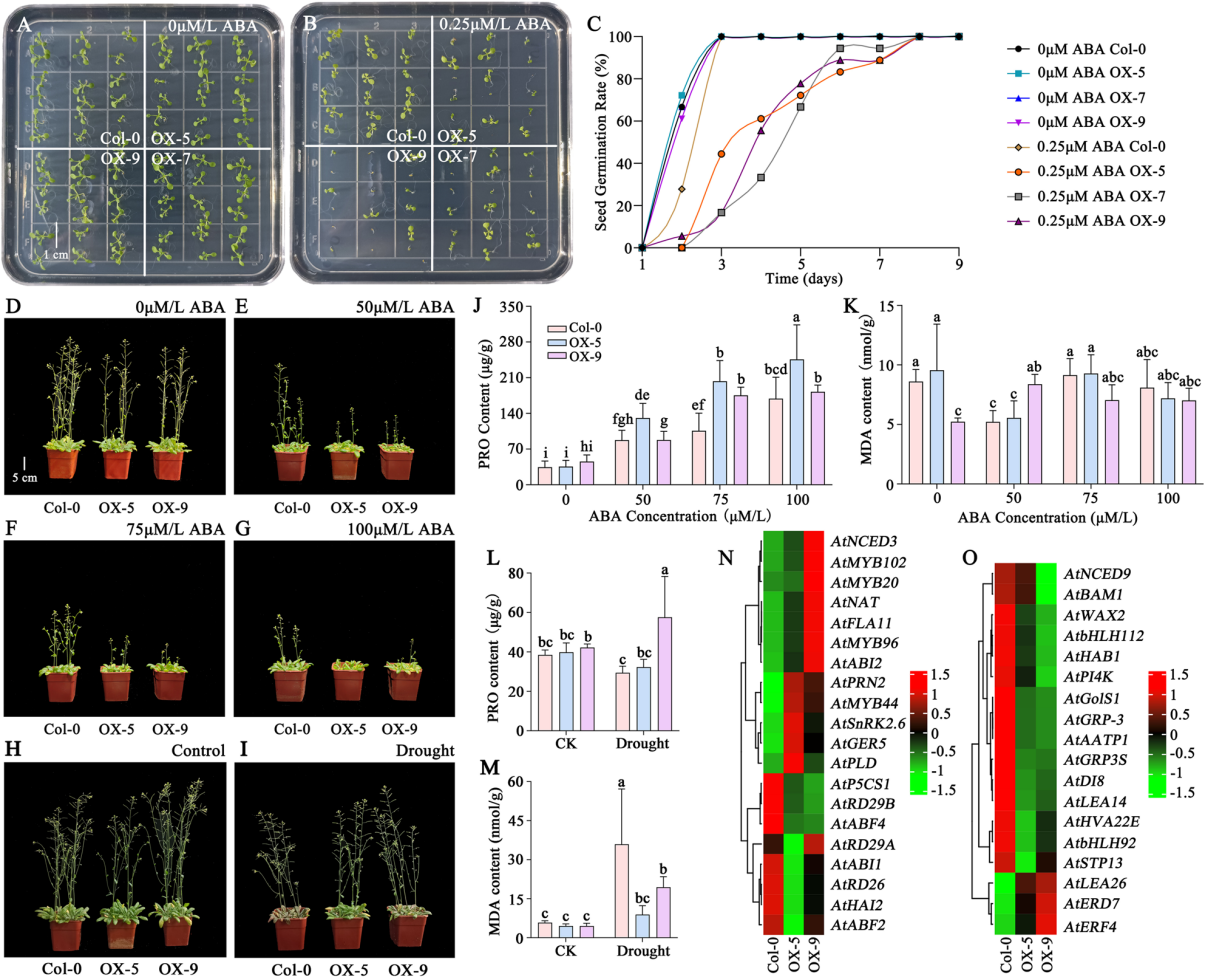
The effect of exogenous ABA and drought on *SiLRL1* heterologous expression of Arabidopsis. **A** and **B**. Phenotype of lines grown on medium with exogenous ABA supply. Seeds were grown on 1/2 MS medium without or with exogenous ABA for 13 days. Scale bar, 1 cm. Three independent experiments were performed with similar trends. **C**. Seed germination rate in Col-0 and three *SiLRL1* heterologous expression lines of Arabidopsis (OX-5, OX-7, OX-9) grown on medium with exogenous ABA. Values are presented as means±SD from three independent samples. **D**-**G**. Phenotype of Col-0, OX-5 and OX-9 with exogenous ABA of 0 (D), 50 (E), 75 (F) and 100 (G) μM/L for 15 days. H-I. The phenotype of Col-0 and two SiLRL1 heterologous expression lines (OX-5, OX-9) that were exposed to the control (**H**) and drought **I**. J-K. PRO (**J**) and MDA (**K**) content of Col-0, OX-5 and OX-9 with exogenous ABA. L-M. PRO (**L**) and MDA (**M**) content of Col-0, OX-5 and OX-9 under control and drought. The error bars represent the SD of three biological replicates. Lowercase letters indicate significant difference (P<0.05, Tukey’s multiple comparisons test). **N**-**O**. Heatmap of differentially expressed genes related to ABA and drought stress responses in Col-0, OX-5 and OX-9. Expression values (FPKM) were normalized using Z-score

To examine the response to exogenous ABA, seeds of Col-0 and three transgenic lines (OX-5, OX-7, OX-9) were germinated on 1/2 MS medium with or without ABA. Although 0.25 µM ABA delayed germination in all transgenic lines, the final germination percentage was unaffected (Fig. 7A-C). We further assessed ABA sensitivity in one-month-old plants by foliar spraying with ABA solutions (0, 50, 75, and 100 µM/L) for 10 days. Transgenic lines OX-5 and OX-9 displayed greater phenotypic sensitivity to ABA than Col-0, with stronger growth inhibition at higher ABA concentrations (Fig. 7D-G). PRO content was higher in the transgenics and increased significantly with ABA concentration (Fig. 7 J), whereas MDA levels remained stable across treatments (Fig. 7 K).

Given these findings, we hypothesized that *SiLRL1* overexpression enhanced drought tolerance. Under drought stress, Col-0 plants showed pronounced leaf yellowing and wilting after one week, while OX-5 and OX-9 maintained relatively normal growth (Fig. 7H-I). Although MDA levels were similar across genotypes under well-watered conditions, drought stress led to a significant increase in MDA, with Col-0 accumulating markedly more than the transgenic lines (Fig. 7M). PRO content remained comparable under control conditions but was hyper-accumulated specifically in OX-9 following drought (Fig. 7L). RNA-seq analysis revealed substantial expression changes in 20 ABA-responsive genes (e.g., *AtABF4*, *AtRD29B*, *AtNCED3*; Fig. 7 N, Supplementary Table S12) and 18 drought-responsive genes (e.g., *AtERD7*, *AtLEA26*, *AtLEA14*; Fig. 7O, Supplementary Table S12), supporting the role of *SiLRL1* in modulating ABA and drought stress responses.

## Discussion

### Phylogenetic and structural characterization of bHLH transcription factors in foxtail millet

Carotenoids are natural pigments that are abundant in fruits and vegetables. As beneficial compounds, carotenoids can reduce the risk of various diseases such as cancer, eye disorders, and cardiovascular diseases and eliminate peroxide free radicals [64]. Because the human body cannot synthesize carotenoids and depends on limited dietary sources, enriching foods with carotenoids is necessary to increase our intake. Kernel of foxtail millet is golden in color and rich in carotenoids, making it a model crop for studying carotenoid metabolism in monocotyledonous plants.

To further explore the regulatory mechanisms underlying carotenoid accumulation in foxtail millet, we focused on the bHLH transcription factor family, which has been implicated in various metabolic pathways. The classification of bHLH transcription factors in *Arabidopsis thaliana* has undergone significant refinement. Initially, 133 members were categorized into 12 groups (I–XII) in 2003 [65]. This classification was subsequently expanded to 15 groups (I–XV) in 2010 [66], following more comprehensive genomic analyses. Most recently, the system was further extended to 17 groups (I–XVII) in 2024 [63]. In this study, we selected the superfamily sequences of 158 AtbHLHs and 185 SibHLHs (Supplementary Table S3) to construct a phylogenetic tree, rather than the full-length amino acid sequences (Supplementary Table S2), resulting in some differences from the previous reported groups of AtbHLHs [63].

Based on this, we jointly constructed a phylogenetic tree of bHLHs from foxtail millet and *Arabidopsis thaliana*, and divided it into 14 groups (Fig. 1). Most bHLH transcription factors possess two characteristic functional domains: a basic region and an HLH domain. However, further analysis indicates that members of the XI subfamily retain only the HLH domain and lack the DNA-binding basic region (Fig. 2 A). Perhaps due to this structural distinction, the SiLRL1 protein is not localized to the nucleus (Fig. 3E).

### SiLRL1 orchestrates carotenoid biosynthesis and ABA-mediated drought resistance

Haplotype analysis of *SiLRL1* revealed no significant association with kernel color in 406 varieties of foxtail millet (Fig. 2 F), which suggests that kernel color might be controlled by multiple genes with redundant functions, or the effect of *SiLRL1* may be masked by stronger epistatic interactions with other loci. Also, Our comprehensive expression analysis reveals that the 185 *SibHLH* genes display broad but variable expression patterns across diverse tissues (Supplementary Figure S1), indicating substantial functional diversification and roles in multiple aspects of the development of foxtail millet. Notably, *SiLRL1*, a member of the XⅠ subfamily, which shows pronounced tissue-specific expression with significant accumulation in grains (Fig. 3 A and D). This distinct spatial expression profile implies a specialized role for *SiLRL1* in grain development and related physiological processes, potentially influencing grain quality, filling, or maturation. It is noteworthy that the expression level of *SiLRL1* in leaves and root of JG21 at the jointing stage was significantly higher than that at the seedling and heading stages (Fig. 3B). This expression pattern may be closely related to the physiological characteristics of the jointing stage as a critical period for water demand in foxtail millet. The expression peak likely reflects the role of *SiLRL1* in coordinating growth and stress defense. Just as other bHLH transcription factor (bHLH6, bHLH92, bHLH112) [39, 67, 68], *SiLRL1* may respond to drought and ABA signals, activate downstream stress-responsive gene networks, thereby addressing the conflict between high transpiration demand and drought sensitivity during this period.

The heterologous expression of *SiLRL1* in Arabidopsis led to significant alterations in transcription related to kernel color and carotenoid metabolism. Among the key DEGs, *AtCCD4*/*NCED4*, a central enzyme gene in carotenoid cleavage, was notably downregulated in OX-5 and OX-9 (Fig. 4D). This suppression likely reduces carotenoid degradation, suggesting a conserved mechanism by which *SiLRL1* may enhance carotenoid accumulation, thereby potentially influencing kernel color in foxtail millet. KEGG analysis revealed enrichment in metabolic pathways closely associated with color formation, including carotenoid and flavonoid biosynthesis (Fig. 4 F). It is worth noting that, GO enrichment analysis revealed that DEGs were implicated in a range of biological processes, particularly response to abscisic acid and abiotic stimulus (Fig. 4E).

Higher expression of genes such as *AtGGPPS1/2*, *AtGGPS1/3*, *AtIPP1/2* and *AtDXR* across the upstream MVA and MEP pathways of the carotenoid biosynthesis in OX-5 and OX-9 (Fig. 5 A and B) may supply essential isoprenoid precursors [10, 69]. Also, several key genes involved in the carotenoid biosynthesis pathway, such as *AtPSY*, *AtPDS3*, *AtLCYB* and *AtLCYE*, upregulated expression especially in OX-9 (Fig. 5 C), which may increase metabolic flux [70, 71]. On the downstream ABA metabolic branch, the expression of key ABA biosynthetic enzyme genes (*AtNCED2/4/5*) and signaling components genes (*AtABI3/4*) was downregulated, yet the net ABA content was significantly higher in OX-9 (Fig. 5G). So, we thought the heterologous expression of *SiLRL1* may expand the carotenoid precursor pool substrate for the NCED enzymes, potentially overwhelming their reduced transcription levels and subsequently triggering feedback repression of its own biosynthetic and signaling genes.

Previous research showed that ectopic expression of *PtrbHLH66* enhanced drought tolerance of transgenic Arabidopsis. And *SiLRL1* is the homologous gene of *AtLRL1*(*AtbHLH66*) (Figs. 1 and 2G). Therefore, we designed an experiment with exogenous application of 0.25µM/L ABA on 1/2 MS Medium and 50–100µM/L ABA in soil and found that the germination rate of seeds expressing *SiLRL1* was significantly reduced (Fig. 7 A and C), the growth of plants was significantly inhibited (Fig. 7D and G), which indirectly proved that heterologous expression of *SiLRL1* enhanced Arabidopsis sensitivity to ABA. Subsequently, we subjected these plants to drought treatment and found that plants expressing *SiLRL1* were more drought tolerant (Fig. 7H and I) and the MDA content was significantly lower than Col-0 (Fig. 7M). These results suggest that *SiLRL1* may be involved in regulating plant osmotic pressure and alleviating oxidative aging physiology, thereby enhancing the drought resistance of Arabidopsis. Furthermore, we analyzed the expression level of genes related to ABA responses and drought stress (Fig. 7 N and O) and found that *AtNCED3*, which encoded a key, highly regulated dioxygenase that catalyzed the first committed step in ABA biosynthesis [72], up-regulated in OX-9. Therefore, we speculate that SiLRL1 may be a central gene that directly or indirectly regulates the ABA metabolic pathway and drought resistance. Further investigation into *SiLRL1*’s regulatory targets and molecular interactions may provide valuable insights into the genetic control of kernel color and abiotic stress resistance in foxtail millet.

## Conclusion

In this study, we conducted a systematic analysis of the phylogenetic position, structural features, haplotypes, and expression profiles of SiLRL1, a bHLH transcription factor in foxtail millet. Our results revealed that the foxtail millet bHLH family comprises 185 genes, with SiLRL1 classified into subfamily XI. Phylogenetic comparison of LRL1 orthologs from 11 species indicated clear divergence between monocot and dicot lineages, and identified a closest relationship between SiLRL1 (*Setaria italica*) and SvLRL1 (*Setaria viridis*). Expression profiling showed that *SiLRL1* is highly expressed in leaves at the jointing stage, and subcellular localization experiments confirmed its specific presence on the cell membrane. RNA-seq analysis of Arabidopsis heterologously overexpressing *SiLRL1* demonstrated that it modulates the expression of key genes involved in critical biological processes—particularly in carotenoid biosynthesis and abiotic stress responses. Functional assays further revealed that *SiLRL1* overexpression enhances carotenoid and ABA accumulation, and improves drought tolerance and ABA sensitivity in Arabidopsis. Collectively, these findings highlight SiLRL1 as a promising genetic target for simultaneously improving nutritional quality and stress resilience in crops.

## Supplementary Information

Supplementary Material 1. Figure S1: Heatmap of 185 *SibHLHs* expression of different tissues in Yugu1.

Supplementary Material 2. Figure S2: Information of motif sequences and locations of 8 SibHLHs in the XI subfamily.

Supplementary Material 3. Table S1: Functional annotation of 185 *SibHLH* genes.

Supplementary Material 4. Table S2: Protein sequences of the 158 AtbHLH and 185 *SibHLH* genes.

Supplementary Material 5. Table S3: Conserved superfamily domain sequences of 158 AtbHLH and 185 SibHLH proteins.

Supplementary Material 6. Table S4: Information of genes and proteins of the bHLH members of XI subfamily.

Supplementary Material 7. Table S5: Identifiers and protein sequences of LRL1 orthologs from 11 species.

Supplementary Material 8. Table S6: 185 SibHLHs expression of different tissues in Yugu1.

Supplementary Material 9. Table S7: Specific results of *SiLRL1* haplotype analysis from a core collection of 406 conventional foxtail millet varieties.

Supplementary Material 10. Table S8: Summary of RNA-seq read mapping results.

Supplementary Material 11. Table S9: Expression levels and enrichment analysis in multiple databases of DEGs in Col-0, OX-5 and OX-9.

Supplementary Material 12. Table S10: Expression levels and enrichment analysis in multiple databases of genes of carotenoid pathway in Col-0, OX-5 and OX-9.

Supplementary Material 13. Table S11: Primer sequences used in this study.

Supplementary Material 14. Table S12: Table of the correspondence between gene IDs and names for ABA- and drought-responsive genes.

## Data Availability

Data supporting the findings of this work are available within the paper and the Supplementary Tables and Figures. The raw sequence data reported in this paper have been deposited in the Genome Sequence Archive (Genomics, Proteomics & Bioinformatics 2021) in National Genomics Data Center (Nucleic Acids Res 2022), China National Center for Bioinformation/Beijing Institute of Genomics, Chinese Academy of Sciences (GSA: CRA030335) that are publicly accessible at https://ngdc.cncb.ac.cn/gsa. Other relevant materials are available from the corresponding authors on reasonable request.

## Abbreviations

TF: Transcription factor
bHLH: Basic helix-loop-helix
ABA: Abscisic acid
MVA: Mevalonate
MEP: Methylerythritol phosphate
LCYB: Lycopene β-cyclase
LCYE: Lycopene ε-cyclase
PSY: Phytoene synthase
CRTISO: Carotenoid isomerase
ZEP: Zeaxanthin epoxidase
NSY: Neoxanthin synthase
NCED: 9’-cis-neoxanthin dioxygenase
ABA2: Abscisic Acid Deficient 2
ABI5: Abscisic Acid Insensitive 5
HLH: α-helix-loop-α-helix
JG21: Jingu 21
DAP: Days after planting
DL1: Newly emerging leaf
DL2: Expanding leaf
DL3: Fully expanded leaf
Q: Leaf sheath
J: Stem
ASA: Tissue around the shoot apex
G: Root
S1: Initial stage of the millet grain filling period
S3: Middle stage of the millet grain filling period
S5: Late stage of the millet grain filling period
WT: Wild-type
MDA: Malondialdehyde
PRO: Proline
SOPMA: Self-Optimized Prediction Method with Alignment
a*: Red/green value
b*: Yellow/blue value
L*: Lightness value
CDS: Coding sequence
Pfam: Protein family
KOG/COG: Clusters of Orthologous Groups of Proteins
KO: KEGG Ortholog
GO: Gene Ontology
FPKM: Fragments per kilobase of transcript per million mapped reads
FDR: False discovery rate
DEGs: Differentially expressed genes
AACC: American Association of Cereal Chemists
UHPLC: Ultra-high performance liquid chromatography
QC: Quality control
ESI: Electrospray ionization
SIM: Selected ion monitoring
CDD: Conserved Domain Database
Sbs: The single base substitution
Saas: The single amino acid substitution
Num: Number of varieties
PPVED: Plant protein variation effect detector
GA: Gibberellin
SA: Salicylic acid
IAA: Indole-3-acetic acid
JA: Jasmonic acid
JA-Ile: Jasmonic acid-isoleucine
