# Supplementary figures and images for: SiLRL1, a bHLH transcription factor from foxtail millet, promotes carotenoid accumulation and improves drought tolerance
Source: BMC Plant Biol. 2025 Dec 6;26:62. doi: 10.1186/s12870-025-07825-8 (PMC12797406; doi:10.1186/s12870-025-07825-8)

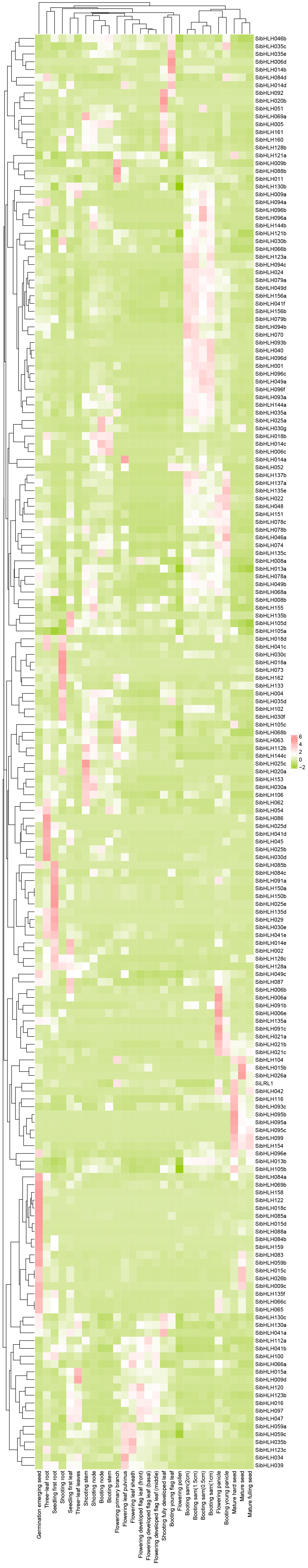

Supplement: Supplementary file 1 — Supplementary Material 1. Figure S1: Heatmap of 185 SibHLHs expression of different tissues in Yugu1. [file 12870_2025_7825_MOESM1_ESM.png]

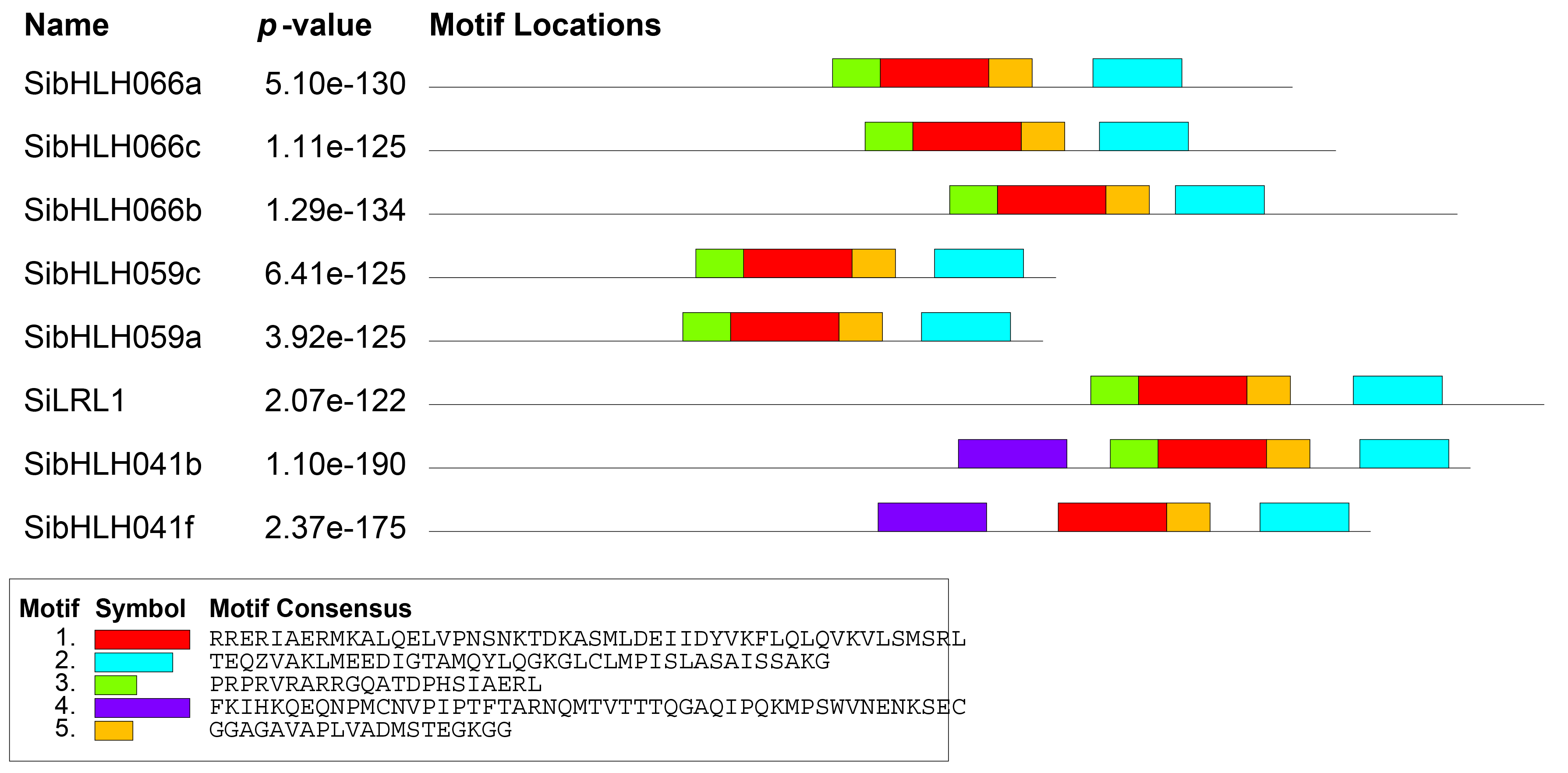

Supplement: Supplementary file 2 — Supplementary Material 2. Figure S2: Information of motif sequences and locations of 8 SibHLHs in the XI subfamily. [file 12870_2025_7825_MOESM2_ESM.png]
