# Supplementary material for: SiLRL1, a bHLH transcription factor from foxtail millet, promotes carotenoid accumulation and improves drought tolerance
Source: BMC Plant Biol. 2025 Dec 6;26:62. doi: 10.1186/s12870-025-07825-8 (PMC12797406; doi:10.1186/s12870-025-07825-8)
Supplement: Supplementary file 13 — Supplementary Material 13. Table S11: Primer sequences used in this study. [file 12870_2025_7825_MOESM13_ESM.docx]

Supplemental Table S11. Primer sequences used in this study

| Primer name | Primer Sequence (5’-3’) |
| --- | --- |
| SiLRL1c-F1 | TACATGGTAGATCTTGGATCCATGGGTGGCTTCGTGGACCC BamHI |
| SiLRL1c-R1 | ACGATCGGGGAAATTCGAGCTCTTAGGCCCTGGACTTCATCT SacI |
| Hyg-F | ACGGTGTCGTCCATCACAGTTTGCC |
| Hyg-R | GGAAGTGCTTGACATTGGGGAGTTT |
| qSiActin-F | TGCTCAGTGGAGGCTCAACA |
| qSiActin-R | GAGCGCAAGTACAGTGTCTGG |
| qSiLRL1-F | TTCACTGGCACATCCTGGTC |
| qSiLRL1-R | ACAGGCTTGGACCTGTTCTG |
| qAtActin-F | GGTGTCATGGTTGGTATGGGTC |
| qAtActin-R | CCTCTGTGAGTAGAACTGGGTGC |
| qAtDXS1-F | CGCAGAGAGAGCTGATGACAA |
| qAtDXS1-R | TGCCTCTGCTTCTGCAATCA |
| qAtDXS2-F | GCTCGCACGTTGTTCAGTTT |
| qAtDXS2-R | TGGCATGAGTCCAGCTTCAG |
| qAtDXS3-F | TGTCCCCACTGGATTACCAA |
| qAtDXS3-R | CGAGAGAAGCGAGTGAGCAT |
| qAtIPP1-F | GTCTGTTCGCGCTTTCTCAG |
| qAtIPP1-R | CATGTCCCACCACACGATCA |
| qAtIPP2-F | ATGGCAAATGGGGAGAGCAT |
| qAtIPP2-R | TTCACCAGCTCCTTCAGCTC |
| qAtBCH1-F | TGCTTTGGCGCCGGGTTA |
| qAtBCH1-R | CTTGTCTGTGTGATGTAGCTGGTG |
| qAtBCH2-F | GCTGTGATGTCGAGCTTTGG |
| qAtBCH2-R | AAAACTCCATCCCAACGGCA |
| qAtCCD1-F | CTTCCACGCTATGCCAAGGA |
| qAtCCD1-R | ACGACAAGTGATGAGGACGAC |
| qAtCRTISO-F | CTGCGTGGGGGATAGTTGTT |
| qAtCRTISO-R | CGAGTGTCCTTAGCCAACCA |
| qAtCYP97A3-F | TCGAAGTCCTCTGCATTGGG |
| qAtCYP97A3-R | GGTCCTCCACCGAAAGGTAA |
| qAtCYP97C1-F | GGCTCACAACACTCACTCGT |
| qAtCYP97C1-R | AGCGAGACGGTAAATGGGTC |

Supplemental Table S1 Continued. Primer sequences

| Primer name | Primer Sequence (5’-3’) |
| --- | --- |
| qAtLCYB-F | TGGCGGTTTCAACATTTCCG |
| qAtLCYB-R | CACCGCTACACAACTCTCACT |
| qAtLCYE-F | TCATCCCTCAGTTTCATGGGTT |
| qAtLCYE-R | AGCGCTACCACTCACTACAC |
| qAtPDS-F | TACTGCTGGTCCTTTGCAGG |
| qAtPDS-R | AAAGGCTTAGCAGGACGAGG |
| qAtPSY-F | CTTGTGGATGGGCCAAATGC |
| qAtPSY-R | CTGTATCAGCGAGAGCAGCA |
| qAtVDE-F | TGGGAGATCCTCACGTCCTT |
| qAtVDE-R | GGAACGCGCAAGCTAAAACA |
| qAtZDS-F | TCGAGGGAGTACCTGTGGTT |
| qAtZDS-R | AGATCCGCAAAGCACGAGAA |
| qAtZEP-F | TCGAAGATGCATGCTCGTGT |
| qAtZEP-R | TGTCGGACGATCTAAACCGC |
| qAtZ-ISO-F | CGACATCCTCAGATGGTTGGA |
| qAtZ-ISO-R | CATTCCACGCCCCAAACAAA |
| qAtNCED2-F | AACCTCACCGTCGAAGGAAC |
| qAtNCED2-R | GTGAACCATTCCGTCTCCGT |
| qAtNCED3-F | AGCTAACCCACTTCACGAGC |
| qAtNCED3-R | GATGGCTTTGGGGAAAACCG |
| qAtNCED4-F | AGCGTCGTTTTGATTGCACC |
| qAtNCED4-R | CCTCGCTGAGATCGGATGAC |
| qAtNCED5-F | ACGCCGTAAACTCTCTGCAA |
| qAtNCED5-R | GGTTCCAACGGGAAGTGTCT |
| qAtNCED6-F | GGCTACGATGCTCGACAAGA |
| qAtNCED6-R | AACCGGAGCGAAGTTACCTG |
| qAtNCED9-F | TAAACCCGGTGCAGAGAACC |
| qAtNCED9-R | AGTTCCCGGCTATTTGGACG |
